# Supplementary material for: How to Educate the Public about Dental Trauma—A Scoping Review
Source: Int J Environ Res Public Health. 2022 Feb 21;19(4):2479. doi: 10.3390/ijerph19042479 (PMC8872546; doi:10.3390/ijerph19042479)
Supplement: Supplementary file 1 [file ijerph-19-02479-s001.zip › ijerph-1560805-supplementary.pdf]

Table S1. Full summary of studies focused on education in TDI (* data not included into analysis).

Authors, Year, Country	Modality	Study Design/ Survey Instrument	Sample	Intervention	Term of Observation	Major Findings
Kahabuka et al. [19] 2003 Tanzania	a seminar *vs.* a guidelines sent by email	post-test control group design<br><br>questionnaire adapted from previous studies	teachers<br><br>cluster sampling/ randomization at the level of school<br><br>Intervention groups:<br>mailed guidelines group =185<br>seminar group =272<br><br>controls =198	a brochure sent via email to the headmaster of the school with the request to convey the information to all teachers<br><br>a seminar for two selected teachers, school headmaster and teacher responsible for health affairs from each school + a brochure + a request to disseminate the information to their colleagues<br><br>presented topic: treatment of avulsed teeth<br><br>controls - no intervention	6 month	the seminar was more effective than the guidance sent by e-mail in the teacher's attitude towards the replantation of the avulsed tooth and in the choice of an appropriate means of transport for the avulsed tooth (p<0.0005)<br><br>in some aspects (cleaning method or type of liquid they would use to clean a soiled avulsed tooth), no differences were observed between the three groups
McIntyre JD at al. [20] 2006 USA	a pamphlet *vs.* the pamphlet and 10-minute lecture	pre-test-post-test study design<br><br>questionnaire developed by authors	public elementary school teachers, teacher's assistants, nurses<br><br>cluster sampling/ randomization at the level of school<br><br>Intervention groups:	Intervention group P: pamphlet available for 20 minutes<br>Intervention group P+L: pamphlet available for 20 minutes followed by the 10-minute lecture with Q&A session<br><br>presented topic: treatment of avulsed teeth	3 months<br><br>questionnaire to be completed at the baseline, immediately after intervention and three months after intervention	both interventions were effective in increasing TTKS between baseline and follow-up (C vs P p=0.015, C vs P+L p=0.002).<br><br>a 10-minute TDI lecture provided no additional benefit compared with an educational booklet (P vs P + L, NS).

			pamphlet (P) = 37<br>pamphlet + lecture (P+L)= 29<br><br>controls (C) = 45	controls - no intervention		
Holan et al. [21]<br>2006<br>Israel	a seminar	pre-test post-test study design<br><br>questionnaire developed by authors	physical education teachers<br><br>cluster sampling<br><br>baseline =126<br>follow up = 100 (including 70 teachers attending the seminar and 30 not attenders)	seminar + question and answer session on the topic presented at the biennial meeting of physical education teachers<br><br>topic presented: tooth avulsion	10 months<br><br>pre-test carried out 6 months prior to intervention	the percentage of teachers who gave correct answers was significantly higher in the second questionnaire than in the first one (p<0.001)<br><br>no difference in the percentage of teachers who gave correct answers in the second questionnaire among seminar participants and non-participants<br><br>"contamination effect" - sharing and disseminating information provided at the seminar
Al-Asfor A et al. [22]<br>2008<br>Kuwait	a lecture	one-group pre-test post-test study design<br><br>questionnaire developed by authors	teachers<br><br>cluster sampling<br><br>intervention group = 43	30-minute lecture + question-and-answer session held at the school<br><br>topic presented: avulsion and replantation of a tooth	after intervention<br><br>pre-test carried out 6 months prior to intervention	Increase in knowledge in all surveyed areas from low to sufficient<br><br>problems with reaching the whole population
Al-Asfor A, Andersson L [23]<br>2008<br>Kuwait	a leaflet	post-test control group design	parents<br><br>cluster sampling/	a leaflet<br><br>presented topic: tooth avulsion and replantation	1 week	subjects reading a leaflet got better scores than controls (data were not statistically analysed)

		questionnaire developed by authors	randomization at the level of participant<br><br>intervention group = 85<br><br>controls =75	controls - no intervention		for an extra-oral time the knowledge gain was not sufficient
Lieger O et al. [24]<br>2009<br>Switzerlan d	a poster	post-test control group design<br><br>self-administrated questionnaire send by mail to schools (10 questionnaires /school)<br><br>questionnaire form adapted from previous studies	teachers<br><br>cluster sampling<br><br>intervention group (teachers working in the area with poster distribution) = 185<br><br>controls (teachers working in the area without poster distribution) = 326	a poster sent to all schools from Canton of Bern<br><br>presented topics: the emergency management of injured teeth<br><br>controls – no intervention	5 years	teachers who worked in the area where the posters were distributed had more knowledge about the management of different types of dental injuries (data not statistically analysed)
Frujeri M de L, Costa ED Jr. [25]<br>2009<br>Brazil	a lecture	pre-test post-test study design<br><br>questionnaire adapted from previous studies	different professionals<br><br>cluster sampling<br><br>intervention groups:<br>elementary school teachers =102,	a 40 minute lecture + Q&A session<br><br>presented topics: tooth avulsion	2 months	in each group the percentage of correct answers was significantly higher after the lecture ($p<0.0001$ in teachers, physical education specialists and bank employees and $p<0.01$ in paediatricians)

			physical education professionals = 124, bank employees = 103, dentists* = 100, paediatricians = 50			
Levin et al. [26]<br>2010<br>Israel	a lecture	post-test control group design<br><br>questionnaire adapted from previous studies	18-year-old men military recruits<br><br>cluster sampling/ randomization at the level of participant<br><br>intervention group = 199<br>controls = 137	a 60-minute lecture<br><br>presented topics: body facial and oral injuries and their first-aid management<br><br>controls - no intervention	immediately after intervention	the intervention group presented better knowledge than controls ($p<0.001$)<br><br>a single lecture was not sufficient to deliver complete knowledge
Karande N et al. [27]<br>2012<br>India	a lecture	one-group pre-test post-test study design<br><br>multiple choice questionnaire developed by authors	teachers<br><br>cluster sampling<br><br>Intervention group = 216	a lecture<br><br>presented topics: emergency management of dentoalveolar injuries	3 months	improvements in knowledge were observed in the areas studied: TDI epidemiology, first aid in tooth avulsion and tooth fracture (no statistical analysis presented)<br><br>the level of knowledge on oral prostheses remained low
Arikan and Sònmez [28]<br>2012<br>Turkey	a leaflet	one-group pre-test post-test study design	teachers<br><br>cluster sampling	a leaflet<br><br>presented topics: traumatic dental injuries and their emergency management	1 month	teachers' knowledge of TDI increased significantly ($p = 0.0001$) after distribution of the information leaflet

		questionnaire developed by authors	intervention group = 450			
Skapetis et al. [29] 2012 Australia	an interactive and multimodal workshop	one-group pre-test post-test study design<br><br>questionnaire developed by the authors	physicians, nurse practitioners, medical students recruited<br><br>cluster sampling<br><br>intervention group: at the baseline = 242<br>at the follow up = 181	workshops (lecture, video presentation, discussion, case study, models)<br><br>presented topics: dental nomenclature, dental anaesthesia, effective emergency treatment for common dental trauma, intraoral haemorrhage, dental infections	6 months	the self-reported improvement in proficiency on dental emergency management including avulsion and dental trauma ($p<0.001$)
Baginska and Wilczynska -Borawska [30] 2012 Poland	a lecture	post-test control group design<br><br>questionnaire adapted from previous studies	school nurses<br><br>cluster sampling<br><br>intervention group = 38<br><br>controls = 12	a lecture conducted two years prior to survey<br><br>presented topics: management of TDI<br><br>controls - no intervention	2 years	a strong correlation between the level of knowledge and the participation in the lecture on dental trauma management ($p<0.01$)
Emerich et al. [31] 2013 Poland	a lecture + an additional task	pre-test-post-test control group design<br><br>questionnaire form (three different scenarios of TDI)<br>adapted from previous studies	physical education students<br><br>cluster sampling/ randomization at the level of participant<br><br>intervention group = 49	intervention group: a 30 minute lecture + an additional task of preparing a presentation on dental trauma to retain knowledge<br><br>controls: a 30 minute lecture + an additional task of preparing a presentation on different topic	1 year	a lecture was sufficient to introduce basic knowledge on dental trauma ($p<0.001$)<br><br>the effect was long-lasting<br><br>the additional task (preparing of the presentation) had no influence on the long term retention of knowledge (NS)

			controls = 50	presented topics: permanent dentition injuries		
Soubra BN and Debs NN [32] 2013 Lebanon	an audio and visual methods	1st intervention one-group pre-test post-test study design<br><br>2nd intervention post-test control group design<br><br>two-questions test developed by authors	pupils (8-11 years old)<br><br>cluster sampling/ randomization – not specified<br><br>1st intervention Intervention group = 172<br><br>2nd intervention group A = 86 group B = 86	1st intervention: a cartoon movie<br><br>2nd intervention: visual *vs.* audio modes (group A - a cartoon twice: once without verbal commentary and once with comments, group B listen to the story twice, second time simultaneously with cartoon)<br><br>presented topics: tooth avulsion	1st intervention: 3 months<br><br>2nd intervention: immediately after intervention	1st intervention watching cartoon movie resulted in increasing the knowledge ($p<0.001$)<br><br>2nd intervention a message repetition increased the number of correct answers (group A $p<0.001$, group B $p=0.03$)<br><br>both methods of providing information (two projections and one reading vs. two readings and one projection) were similarly effective (NS)<br><br>telling a story has a greater impact than a silent movie ($p<0.001$)<br><br>children preferred cartoon than story
Ghaderi et al. [33] 2013 Iran	a leaflet	post-test control group design<br><br>questionnaire adapted from previous studies	parents<br><br>cluster sampling/ randomization at the level of participant<br><br>intervention group =75	a leaflet + an assistance of a senior dental student<br><br>controls - no intervention<br><br>presented topics: tooth avulsion	1 week	the leaflet + the assistance of knowledgeable dental staff improved the level of parents' knowledge (data were not statistically analysed)

			controls = 75			
Pujita et al. [34]<br>2013<br>India	a lecture	pre-test-post-test study design<br><br>questionnaire developed by authors	teachers<br><br>cluster sampling<br><br>intervention group = 1000 (urban group = 500 and rural group = 500)	a 30 minute lecture + Q&A session<br><br>presented topics: management of TDI	3 months	an improvement in teachers' knowledge was observed (data were not statistically analysed)<br><br>according to the authors, the knowledge gain was below the expected level
Young C et al. [35]<br>2013<br>Hong Kong	a poster	cluster randomised controlled trial<br><br>post-test control group design<br><br>questionnaire adapted from previous study	teachers<br><br>cluster sampling/ randomization at the level of school<br><br>intervention group = 196<br><br>controls = 212	a poster (three copies available for teachers for two weeks)<br><br>presented topics: dental trauma management<br><br>controls - no intervention	up to 1 week	the posters improved the knowledge about dental trauma management in those subjects who had not previously learned about dental emergencies from other sources than first aid training ($p<0.0001$)
Young C et al. [36]<br>2014<br>Hong Kong	a poster	cluster randomised controlled trial<br><br>post-test control group design<br><br>questionnaire adapted from previous study	pupils (11-20 years)<br><br>cluster sampling/ randomization at the level of school<br><br>intervention group = 364<br><br>controls = 303 pupils	a poster (three copies available for pupils for two weeks)<br><br>presented topics: dental trauma management<br><br>controls - no intervention	up to 1 week	a two week display of the educational poster improved the level of pupils' knowledge on the emergency management of dental trauma in comparison to the control group ($p=0.04$)<br><br>Respondents have problems with acquisition of some information from the poster

Raoof et al. [37]<br>2014<br>Iran	a multimodal educational interventio n (lecture + poster)	one-group pre-test post-test design<br><br>questionnaire adapted from previous study	health teachers<br><br>cluster sampling<br><br>intervention group =38	a 120 minute lecture + Q&A session + an informative poster<br><br>presented topics: general concepts of dental trauma and emergency handling of crown fractures and avulsion injuries	3 years<br><br>questionnaire to be completed at the baseline, immediately after intervention and three year after intervention	a multifaceted educational programme resulted in significant short- and long-term improvements in TDI emergency management knowledge ($p<0.017$)<br><br>participants' self-assessment of their ability to take appropriate action when needed increased significantly ($p=0.0001$)
Ghadimi et al [38]<br>2014<br>Iran	a poster	pre-test post-test control group design<br><br>questionnaire developed by the authors including cases of TDI	school health teachers<br><br>cluster sampling/ randomization at the level of school<br><br>intervention group = 30<br><br>controls = 10	an educational poster<br><br>presented topics: management of TDI<br><br>controls: no intervention	1 month	the percentage of correct answers in the test group increased significantly in the test group one month after the intervention ($p<0.001$)

Grewal et al. [39] 2015 India	a multimodal educational intervention (flip cards + poster + interactive sessions and a lecture)	pre-test post-test study design<br><br>two questionnaires developed by authors: one for parents and school teachers and second for children aged 7–12 years	school teachers/sport coaches, pupils (7-12 years old), parents<br><br>cluster sampling<br><br>intervention groups:<br>pupils = 200<br>parents = 200<br>teachers/sports coaches = 189	1st step: flip cards + a poster placed in the medical room at school<br><br>2nd step: reinforcement session by an interactive sessions and a re-orientation lecture<br><br>presented topics: tooth avulsion	6 months<br><br>questionnaire to be completed two months before intervention, one month after intervention and six months after intervention	an improvement in the knowledge of teachers, parents and pupils about tooth avulsion was observed (p<0.001)
Perazzo et al. [40] 2015 Brazil	a lecture	one-group pre-test post-test study design<br><br>questionnaire adapted from previous studies	firefighters<br><br>cluster sampling<br><br>intervention group = 90	a lecture + a Q&A session<br><br>presented topics: tooth avulsion	6 months<br><br>questionnaire to be completed at the baseline, immediately after intervention and six months after intervention	the intervention resulted in significant short- and long-term improvements in knowledge of tooth avulsion (p<0.001)<br><br>attitudes of paramedics towards tooth replantation decreased over time (p=0.001)<br><br>the need for repeated interventions was raised
Iskander et al. [41] 2016 USA	a mobile application *vs.* a poster	post-test study design<br><br>written questionnaire developed by authors and oral interview questions	parents<br><br>cluster sampling/ randomization at the level of participant<br><br>intervention groups:	1st intervention: one educational tool (mobile application or poster) was used<br><br>2nd intervention: subjects received the other tool to find the participants' preferences	immediately after intervention	1st intervention:<br>both a poster and a mobile healthcare application are effective in delivering TDI information, but respondents using an application significantly more likely immediate replantation as a best option in tooth avulsion (p<0.007)

		conducted by the researcher	mobile application = 45<br>poster = 44	presented topics: management of TDI		2nd intervention:<br>the participants preferred the tool they were given first
Cruz-da-Silva BR et al. [42]<br>2016<br>Brazil	a multimodal educational intervention (lecture + pamphlet)	one-group pre-test post-test study design<br><br>questionnaire developed by authors	non-dental health professional involved in the emergency care service<br><br>cluster sampling<br><br>intervention group = 73 (11 physicians, 41 nurses and 21 paramedics)	a 40 minute lecture + a pamphlet<br><br>presented topics: tooth avulsion	6 months<br><br>questionnaire to be completed at the baseline, immediately after intervention and six months after intervention	improvement in nurses' attitudes towards tooth replantation was observed both immediately after the intervention and six months later ($p<0.001$)
Taranath M et al. [43]<br>2017<br>India	a Power Point presentation	one-group pre-test post-test design<br><br>questionnaire adapted from previous study	school teachers<br><br>cluster sampling<br><br>intervention group = 214	a PowerPoint presentation and demonstration of the first aid procedures in tooth avulsion using a typodont<br><br>presented topics: tooth avulsion	1 month	the knowledge and the attitude towards the management of tooth avulsion were raised ($p<0.0001$)
Al-Musawi A et al. [44]<br>2017<br>Kuwait	a lecture *vs.* a lecture + access to the Dental Trauma App *vs.* access to the Dental Trauma App only	pre-test post-test study design<br><br>questionnaire adapted from previous study	female teacher<br><br>convenience sampling<br><br>intervention groups:<br>Group 1: lecture = 32	a 30-minute lecture<br><br>a lecture + access to the Dental Trauma App when being tested<br><br>no formal instructions + access to the Dental Trauma App when being tested	immediately after intervention	all methods were effective means of knowledge transfer about tooth avulsion (group 1 $p=0.001$, group 2 $p=0.019$, group 3 $p=0.000$)<br><br>the Dental Trauma App alone was superior to a lecture-based delivery of information (the active learning component) ($p<0.001$)

			Group 2: lecture + App = 27<br><br>Group 3: App = 28	presented topics: tooth avulsion		
Yordi et al. [45]<br>2017<br>Lebanon	a multimodal educational intervention (Power Point presentation + brochure + poster)	pre-test post-test study design<br><br>questionnaire adapted from previous study	Teachers<br><br>cluster sampling/ randomization at the level of school<br><br>intervention groups = 300<br><br>controls = 300	a Power point presentation + a brochure + a poster<br><br>presented topics: management of TDI<br><br>controls - no intervention	6 months	the educational program using more than one modality proved to be effective in improving teachers' knowledge (p<0.001)<br><br>the improvement persisted over the time
Al Sari et al. [46]<br>2018<br>United Arab Emirates	a multimodal educational intervention (workshop + poster)	one-group pre-test post-test study design<br><br>questionnaire adapted from previous study	school nurses and physical education teachers<br><br>cluster sampling<br><br>Intervention group = 68	a workshop + a poster<br><br>presented topics: management of TDI	3 months<br><br>questionnaire to be completed at the baseline (A), immediately after intervention (B) and three months after intervention (C)	a significant improvement in the score of knowledge between survey A and B and A and C was observed (p<0.01)<br><br>the knowledge gain sustained after 3 months
Nagata et al. [47]<br>2018<br>Brazil	a lecture	pre-test post-test study design<br><br>questionnaire developed by authors	health courses' students<br><br>cluster sampling<br><br>intervention groups: dentistry students* = 70	a 40-minute lecture + a Q&A session<br><br>presented topics: avulsion and crown fractures	immediately after intervention	there was a significant improvement in knowledge of dental emergencies following the educational intervention (p<0.001)

			nursing students = 33 speech therapy students = 22			
Nashine et al. [48]<br>2018<br>India	an audio vs. audio-visual aids (not specified)	one-group pre-test post-test study design<br><br>questionnaire developed by authors	teachers<br><br>two-stage cluster sampling<br><br>Intervention group = 158<br><br>Participants were divided into audio and audio-visual groups (number not specified)	an intervention through audio or audio-visual aids (not specified)<br><br>presented topics: management of TDI	immediately after intervention	an improvement in knowledge and attitude was observed for whole group (data were not statistically analysed)<br><br>no difference was found with regard to audio and audio-visual aids (NS)
Niviethitha et al. [49]<br>2018<br>India	an interactive educational DVD video	one-group pre-test post-test study design<br><br>questionnaire adapted from previous study	teachers<br><br>randomisation at the level of school and participant<br><br>intervention group =301	an interactive educational DVD video (7-8 minutes)<br><br>presented topics: management of TDI	immediately after intervention	significant improvement in knowledge of TDI management (p<0.0001)<br><br>increase in participants' attitude in dealing with tooth avulsion (p<0.0001)
Razeghi et al.<br>2019 [50]<br>Iran	a leaflet vs. a lecture	cluster randomized controlled trial<br><br>pre-test post-test study design<br><br>a questionnaire	teachers<br><br>cluster sampling/ randomisation at the level participant<br><br>intervention groups:<br>leaflet = 154	a leaflet<br><br>a 45-minute lecture<br><br>presented topics: management of TDI	6 months<br><br>questionnaire to be completed at the beginning, one month after the end of the intervention and six months after the end of the intervention	knowledge gains were observed in both intervention groups (p<0.001)<br><br>no statistically significant difference in mean knowledge and self-practice scores between the two intervention groups (NS)<br><br>self-practice was weakly correlated with knowledge

		developed by authors	lecture = 138			
Authors, Year, country	Modality	Study design/ Survey instrument	Sample	Intervention	Term of observation	Major findings
Kahabuka et al. [19] 2003 Tanzania	a seminar *vs.* a guidelines sent by email	post-test control group design<br><br>questionnaire adapted from previous studies	teachers<br><br>cluster sampling/ randomization at the level of school<br><br>Intervention groups:<br>mailed guidelines group =185<br>seminar group =272<br><br>controls =198	a brochure sent via email to the headmaster of the school with the request to convey the information to all teachers<br><br>a seminar for two selected teachers, school headmaster and teacher responsible for health affairs from each school + a brochure + a request to disseminate the information to their colleagues<br><br>presented topic: treatment of avulsed teeth<br><br>controls - no intervention	6 month	the seminar was more effective than the guidance sent by e-mail in the teacher's attitude towards the replantation of the avulsed tooth and in the choice of an appropriate means of transport for the avulsed tooth ($p<0.0005$)<br><br>in some aspects (cleaning method or type of liquid they would use to clean a soiled avulsed tooth), no differences were observed between the three groups
McIntyre JD at al. [20] 2006 USA	a pamphlet *vs.* the pamphlet and 10-minute lecture	pre-test-post-test study design<br><br>questionnaire developed by authors	public elementary school teachers, teacher's assistants, nurses<br><br>cluster sampling/ randomization at the level of school	Intervention group P: pamphlet available for 20 minutes<br>Intervention group P+L: pamphlet available for 20 minutes followed by the 10-minute lecture with Q&A session	3 months<br><br>questionnaire to be completed at the baseline, immediately after intervention and three months	both interventions were effective in increasing TTKS between baseline and follow-up (C vs P $p=0.015$, C vs P+L $p=0.002$).<br><br>a 10-minute TDI lecture provided no additional benefit

			Intervention groups: pamphlet (P) = 37 pamphlet + lecture (P+L)= 29<br><br>controls (C) = 45	presented topic: treatment of avulsed teeth<br><br>controls - no intervention	after intervention	compared with an educational booklet (P vs P + L, NS).
Holan et al. [21] 2006 Israel	a seminar	pre-test post-test study design<br><br>questionnaire developed by authors	physical education teachers<br><br>cluster sampling<br><br>baseline =126 follow up = 100 (including 70 teachers attending the seminar and 30 not attenders)	seminar + question and answer session on the topic presented at the biennial meeting of physical education teachers<br><br>topic presented: tooth avulsion	10 months<br><br>pre-test carried out 6 months prior to intervention	the percentage of teachers who gave correct answers was significantly higher in the second questionnaire than in the first one (p<0.001)<br><br>no difference in the percentage of teachers who gave correct answers in the second questionnaire among seminar participants and non-participants<br><br>"contamination effect" - sharing and disseminating information provided at the seminar
Al-Asfor A et al. [22] 2008 Kuwait	a lecture	one-group pre-test post-test study design<br><br>questionnaire developed by authors	teachers<br><br>cluster sampling<br><br>intervention group = 43	30-minute lecture + question-and-answer session held at the school<br><br>topic presented: avulsion and replantation of a tooth	after intervention<br><br>pre-test carried out 6 months prior to intervention	Increase in knowledge in all surveyed areas from low to sufficient<br><br>problems with reaching the whole population

Al-Asfor A, Andersson L [23]<br>2008<br>Kuwait	a leaflet	post-test control group design<br><br>questionnaire developed by authors	parents<br><br>cluster sampling/ randomization at the level of participant<br><br>intervention group = 85<br><br>controls =75	a leaflet<br><br>presented topic: tooth avulsion and replantation<br><br>controls - no intervention	1 week	subjects reading a leaflet got better scores than controls (data were not statistically analysed)<br><br>for an extra-oral time the knowledge gain was not sufficient
Lieger O et al. [24]<br>2009<br>Switzerlan d	a poster	post-test control group design<br><br>self-administrated questionnaire send by mail to schools (10 questionnaires /school)<br><br>questionnaire form adapted from previous studies	teachers<br><br>cluster sampling<br><br>intervention group (teachers working in the area with poster distribution) = 185<br><br>controls (teachers working in the area without poster distribution) = 326	a poster sent to all schools from Canton of Bern<br><br>presented topics: the emergency management of injured teeth<br><br>controls – no intervention	5 years	teachers who worked in the area where the posters were distributed had more knowledge about the management of different types of dental injuries (data not statistically analysed)
Frujeri M de L, Costa ED Jr. [25]<br>2009<br>Brazil	a lecture	pre-test post-test study design<br><br>questionnaire adapted from	different professionals<br><br>cluster sampling<br><br>intervention groups:	a 40 minute lecture + Q&A session<br><br>presented topics: tooth avulsion	2 months	in each group the percentage of correct answers was significantly higher after the lecture ($p<0.0001$ in teachers, physical education specialists and bank employees and $p<0.01$ in paediatricians)

		previous studies	elementary school teachers =102, physical education professionals = 124, bank employees = 103, dentists* = 100, paediatricians = 50			
Levin et al. [26] 2010 Israel	a lecture	post-test control group design<br><br>questionnaire adapted from previous studies	18-year-old men military recruits<br><br>cluster sampling/ randomization at the level of participant<br><br>intervention group = 199 controls = 137	a 60-minute lecture<br><br>presented topics: body facial and oral injuries and their first-aid management<br><br>controls - no intervention	immediately after intervention	the intervention group presented better knowledge than controls (p<0.001)<br><br>a single lecture was not sufficient to deliver complete knowledge
Karande N et al. [27] 2012 India	a lecture	one-group pre-test post-test study design<br><br>multiple choice questionnaire developed by authors	teachers<br><br>cluster sampling<br><br>Intervention group = 216	a lecture<br><br>presented topics: emergency management of dentoalveolar injuries	3 months	improvements in knowledge were observed in the areas studied: TDI epidemiology, first aid in tooth avulsion and tooth fracture (no statistical analysis presented)<br><br>the level of knowledge on oral prostheses remained low
Arikan and Sònmez [28]	a leaflet	one-group pre-test post-test study design	teachers<br><br>cluster sampling	a leaflet	1 month	teachers' knowledge of TDI increased significantly (p =

2012 Turkey		questionnaire developed by authors	intervention group = 450	presented topics: traumatic dental injuries and their emergency management		0.0001) after distribution of the information leaflet
Skapetis et al. [29] 2012 Australia	an interactive and multimodal workshop	one-group pre-test post-test study design<br><br>questionnaire developed by the authors	physicians, nurse practitioners, medical students recruited<br><br>cluster sampling<br><br>intervention group: at the baseline = 242<br>at the follow up = 181	workshops (lecture, video presentation, discussion, case study, models)<br><br>presented topics: dental nomenclature, dental anaesthesia, effective emergency treatment for common dental trauma, intraoral haemorrhage, dental infections	6 months	the self-reported improvement in proficiency on dental emergency management including avulsion and dental trauma ($p<0.001$)
Baginska and Wilczynska-Borawska [30] 2012 Poland	a lecture	post-test control group design<br><br>questionnaire adapted from previous studies	school nurses<br><br>cluster sampling<br><br>intervention group = 38<br><br>controls = 12	a lecture conducted two years prior to survey<br><br>presented topics: management of TDI<br><br>controls - no intervention	2 years	a strong correlation between the level of knowledge and the participation in the lecture on dental trauma management ($p<0.01$)
Emerich et al. [31] 2013 Poland	a lecture + an additional task	pre-test-post-test control group design<br><br>questionnaire form (three different scenarios of TDI)	physical education students<br><br>cluster sampling/ randomization at the level of participant	intervention group: a 30 minute lecture + an additional task of preparing a presentation on dental trauma to retain knowledge<br><br>controls: a 30 minute lecture + an additional task of preparing a presentation on different topic	1 year	a lecture was sufficient to introduce basic knowledge on dental trauma ($p<0.001$)<br><br>the effect was long-lasting<br><br>the additional task (preparing of the presentation) had no influence on the long term retention of knowledge (NS)

		adapted from previous studies	intervention group = 49<br><br>controls = 50	presented topics: permanent dentition injuries		
Soubra BN and Debs NN [32]<br>2013<br>Lebanon	an audio and visual methods	1st intervention one-group pre-test post-test study design<br><br>2nd intervention post-test control group design<br><br>two-questions test developed by authors	pupils (8-11 years old)<br><br>cluster sampling/ randomization – not specified<br><br>1st intervention Intervention group = 172<br><br>2nd intervention group A = 86<br>group B = 86	1st intervention:<br>a cartoon movie<br><br>2nd intervention:<br>visual *vs.* audio modes (group A - a cartoon twice: once without verbal commentary and once with comments, group B listen to the story twice, second time simultaneously with cartoon)<br><br>presented topics: tooth avulsion	1st intervention: 3 months<br><br>2nd intervention: immediately after intervention	1st intervention<br>watching cartoon movie resulted in increasing the knowledge ($p<0.001$)<br><br>2nd intervention<br>a message repetition increased the number of correct answers (group A $p<0.001$, group B $p=0.03$)<br><br>both methods of providing information (two projections and one reading vs. two readings and one projection) were similarly effective (NS)<br><br>telling a story has a greater impact than a silent movie ($p<0.001$)<br><br>children preferred cartoon than story
Ghaderi et al. [33]<br>2013<br>Iran	a leaflet	post-test control group design<br><br>questionnaire adapted from previous studies	parents<br><br>cluster sampling/ randomization at the level of participant	a leaflet + an assistance of a senior dental student<br><br>controls - no intervention<br><br>presented topics: tooth avulsion	1 week	the leaflet + the assistance of knowledgeable dental staff improved the level of parents' knowledge (data were not statistically analysed)

			intervention group =75<br>controls = 75			
Pujita et al. [34]<br>2013<br>India	a lecture	pre-test-post-test study design<br><br>questionnaire developed by authors	teachers<br><br>cluster sampling<br><br>intervention group = 1000 (urban group = 500 and rural group = 500)	a 30 minute lecture + Q&A session<br><br>presented topics: management of TDI	3 months	an improvement in teachers' knowledge was observed (data were not statistically analysed)<br><br>according to the authors, the knowledge gain was below the expected level
Young C et al. [35]<br>2013<br>Hong Kong	a poster	cluster randomised controlled trial<br><br>post-test control group design<br><br>questionnaire adapted from previous study	teachers<br><br>cluster sampling/ randomization at the level of school<br><br>intervention group = 196<br><br>controls = 212	a poster (three copies available for teachers for two weeks)<br><br>presented topics: dental trauma management<br><br>controls - no intervention	up to 1 week	the posters improved the knowledge about dental trauma management in those subjects who had not previously learned about dental emergencies from other sources than first aid training ($p<0.0001$)
Young C et al. [36]<br>2014<br>Hong Kong	a poster	cluster randomised controlled trial<br><br>post-test control group design	pupils (11-20 years)<br><br>cluster sampling/ randomization at the level of school<br><br>intervention group = 364	a poster (three copies available for pupils for two weeks)<br><br>presented topics: dental trauma management<br><br>controls - no intervention	up to 1 week	a two week display of the educational poster improved the level of pupils' knowledge on the emergency management of dental trauma in comparison to the control group ($p=0.04$)<br><br>Respondents have problems with acquisition of some information from the poster

		questionnaire adapted from previous study	controls = 303 pupils			
Raoof et al. [37] 2014 Iran	a multimodal educational intervention (lecture + poster)	one-group pre-test post-test design<br><br>questionnaire adapted from previous study	health teachers<br><br>cluster sampling<br><br>intervention group =38	a 120 minute lecture + Q&A session + an informative poster<br><br>presented topics: general concepts of dental trauma and emergency handling of crown fractures and avulsion injuries	3 years<br><br>questionnaire to be completed at the baseline, immediately after intervention and three year after intervention	a multifaceted educational programme resulted in significant short- and long-term improvements in TDI emergency management knowledge ($p<0.017$)<br><br>participants' self-assessment of their ability to take appropriate action when needed increased significantly ($p=0.0001$)
Ghadimi et al [38] 2014 Iran	a poster	pre-test post-test control group design<br><br>questionnaire developed by the authors including cases of TDI	school health teachers<br><br>cluster sampling/ randomization at the level of school<br><br>intervention group = 30<br><br>controls = 10	an educational poster<br><br>presented topics: management of TDI<br><br>controls: no intervention	1 month	the percentage of correct answers in the test group increased significantly in the test group one month after the intervention ($p<0.001$)

Grewal et al. [39] 2015 India	a multimodal educational intervention (flip cards + poster + interactive sessions and a lecture)	pre-test post-test study design<br><br>two questionnaires developed by authors: one for parents and school teachers and second for children aged 7–12 years	school teachers/sport coaches, pupils (7-12 years old), parents<br><br>cluster sampling<br><br>intervention groups:<br>pupils = 200<br>parents = 200<br>teachers/sports coaches = 189	1st step: flip cards + a poster placed in the medical room at school<br><br>2nd step: reinforcement session by an interactive sessions and a re-orientation lecture<br><br>presented topics: tooth avulsion	6 months<br><br>questionnaire to be completed two months before intervention, one month after intervention and six months after intervention	an improvement in the knowledge of teachers, parents and pupils about tooth avulsion was observed (p<0.001)
Perazzo et al. [40] 2015 Brazil	a lecture	one-group pre-test post-test study design<br><br>questionnaire adapted from previous studies	firefighters<br><br>cluster sampling<br><br>intervention group = 90	a lecture + a Q&A session<br><br>presented topics: tooth avulsion	6 months<br><br>questionnaire to be completed at the baseline, immediately after intervention and six months after intervention	the intervention resulted in significant short- and long-term improvements in knowledge of tooth avulsion (p<0.001)<br><br>attitudes of paramedics towards tooth replantation decreased over time (p=0.001)<br><br>the need for repeated interventions was raised
Iskander et al. [41] 2016 USA	a mobile application *vs.* a poster	post-test study design<br><br>written questionnaire developed by authors and oral interview questions	parents<br><br>cluster sampling/ randomization at the level of participant<br><br>intervention groups:	1st intervention: one educational tool (mobile application or poster) was used<br><br>2nd intervention: subjects received the other tool to find the participants' preferences	immediately after intervention	1st intervention:<br>both a poster and a mobile healthcare application are effective in delivering TDI information, but respondents using an application significantly more likely immediate replantation as a best option in tooth avulsion (p<0.007)

		conducted by the researcher	mobile application = 45<br>poster = 44	presented topics: management of TDI		2nd intervention:<br>the participants preferred the tool they were given first
Cruz-da-Silva BR et al. [42]<br>2016<br>Brazil	a multimodal educational intervention (lecture + pamphlet)	one-group pre-test post-test study design<br><br>questionnaire developed by authors	non-dental health professional involved in the emergency care service<br><br>cluster sampling<br><br>intervention group = 73 (11 physicians, 41 nurses and 21 paramedics)	a 40 minute lecture + a pamphlet<br><br>presented topics: tooth avulsion	6 months<br><br>questionnaire to be completed at the baseline, immediately after intervention and six months after intervention	improvement in nurses' attitudes towards tooth replantation was observed both immediately after the intervention and six months later (p<0.001)
Taranath M et al. [43]<br>2017<br>India	a Power Point presentation	one-group pre-test post-test design<br><br>questionnaire adapted from previous study	school teachers<br><br>cluster sampling<br><br>intervention group = 214	a PowerPoint presentation and demonstration of the first aid procedures in tooth avulsion using a typodont<br><br>presented topics: tooth avulsion	1 month	the knowledge and the attitude towards the management of tooth avulsion were raised (p<0.0001)
Al-Musawi A et al. [44]<br>2017<br>Kuwait	a lecture *vs.* a lecture + access to the Dental Trauma App *vs.* access to the Dental Trauma App only	pre-test post-test study design<br><br>questionnaire adapted from previous study	female teacher<br><br>convenience sampling<br><br>intervention groups:<br>Group 1: lecture = 32	a 30-minute lecture<br><br>a lecture + access to the Dental Trauma App when being tested<br><br>no formal instructions + access to the Dental Trauma App when being tested	immediately after intervention	all methods were effective means of knowledge transfer about tooth avulsion (group 1 p=0.001, group 2 p=0.019, group 3 p=0.000)<br><br>the Dental Trauma App alone was superior to a lecture-based delivery of information (the active learning component) (p<0.001)

			Group 2: lecture + App = 27<br><br>Group 3: App = 28	presented topics: tooth avulsion		
Yordi et al. [45]<br>2017<br>Lebanon	a multimodal educational intervention (Power Point presentation + brochure + poster)	pre-test post-test study design<br><br>questionnaire adapted from previous study	Teachers<br><br>cluster sampling/ randomization at the level of school<br><br>intervention groups = 300<br><br>controls = 300	a Power point presentation + a brochure + a poster<br><br>presented topics: management of TDI<br><br>controls - no intervention	6 months	the educational program using more than one modality proved to be effective in improving teachers' knowledge (p<0.001)<br><br>the improvement persisted over the time
Al Sari et al. [46]<br>2018<br>United Arab Emirates	a multimodal educational intervention (workshop + poster)	one-group pre-test post-test study design<br><br>questionnaire adapted from previous study	school nurses and physical education teachers<br><br>cluster sampling<br><br>Intervention group = 68	a workshop + a poster<br><br>presented topics: management of TDI	3 months<br><br>questionnaire to be completed at the baseline (A), immediately after intervention (B) and three months after intervention (C)	a significant improvement in the score of knowledge between survey A and B and A and C was observed (p<0.01)<br><br>the knowledge gain sustained after 3 months
Nagata et al. [47]<br>2018<br>Brazil	a lecture	pre-test post-test study design<br><br>questionnaire developed by authors	health courses' students<br><br>cluster sampling<br><br>intervention groups: dentistry students* = 70	a 40-minute lecture + a Q&A session<br><br>presented topics: avulsion and crown fractures	immediately after intervention	there was a significant improvement in knowledge of dental emergencies following the educational intervention (p<0.001)

			nursing students = 33 speech therapy students = 22			
Nashine et al. [48] 2018 India	an audio vs. audio-visual aids (not specified)	one-group pre-test post-test study design<br><br>questionnaire developed by authors	teachers<br><br>two-stage cluster sampling<br><br>Intervention group = 158<br><br>Participants were divided into audio and audio-visual groups (number not specified)	an intervention through audio or audio-visual aids (not specified)<br><br>presented topics: management of TDI	immediately after intervention	an improvement in knowledge and attitude was observed for whole group (data were not statistically analysed)<br><br>no difference was found with regard to audio and audio-visual aids (NS)
Niviethitha et al. [49] 2018 India	an interactive educational DVD video	one-group pre-test post-test study design<br><br>questionnaire adapted from previous study	teachers<br><br>randomisation at the level of school and participant<br><br>intervention group =301	an interactive educational DVD video (7-8 minutes)<br><br>presented topics: management of TDI	immediately after intervention	significant improvement in knowledge of TDI management (p<0.0001)<br><br>increase in participants' attitude in dealing with tooth avulsion (p<0.0001)
Razeghi et al. 2019 [50] Iran	a leaflet vs. a lecture	cluster randomized controlled trial<br><br>pre-test post-test study design<br><br>a questionnaire	teachers<br><br>cluster sampling/ randomisation at the level participant<br><br>intervention groups: leaflet = 154	a leaflet<br><br>a 45-minute lecture<br><br>presented topics: management of TDI	6 months<br><br>questionnaire to be completed at the beginning, one month after the end of the intervention and six months after the end of the intervention	knowledge gains were observed in both intervention groups (p<0.001)<br><br>no statistically significant difference in mean knowledge and self-practice scores between the two intervention groups (NS)<br><br>self-practice was weakly correlated with knowledge

		developed by authors	lecture = 138			
